# Edge Effect and the Influence of Biotic and Abiotic Factors on Calliphoridae and Mesembrinellidae (Insecta: Diptera) in Três Picos State Park, Brazil

**DOI:** 10.3390/life16040672

**Published:** 2026-04-15

**Authors:** Wellington Thadeu de Alcantara Azevedo, Mariana dos Passos Nunes, Valmíria Moura Leôncio de Albuquerque, Cláudia Soares Santos Lessa, Jeronimo Alencar, Valéria Magalhães Aguiar

**Affiliations:** 1 Departamento de Microbiologia e Parasitologia, Instituto Biomédico, Universidade Federal do Estado do Rio de Janeiro, Rua Frei Caneca, 94, Centro, Rio de Janeiro 20211-040, RJ, Brazil; wellingtontaa@yahoo.com.br (W.T.d.A.A.);; 2 Programa de Pós-Graduação em Biologia Animal, Universidade Federal Rural do Rio de Janeiro, BR 465, Km 07, Seropédica, Rio de Janeiro 23890-000, RJ, Brazil; 3 Programa de Pós-Graduação em Ciências Biológicas (Biodiversidade Neotropical), Universidade Federal do Estado do Rio de Janeiro, Av. Pasteur 458, Urca, Rio de Janeiro 22290-240, RJ, Brazil; 4 Programa de Pós-Graduação em Medicina Tropical, Fundação Oswaldo Cruz (FIOCRUZ), Av. Brasil, 4365, Rio de Janeiro 21040-900, RJ, Brazil; 5 Laboratório de Diptera, Instituto Oswaldo Cruz, Manguinhos, Rio de Janeiro 21040-360, RJ, Brazil

**Keywords:** anthropization, environmental entomology, biomonitors, conservation, forensic entomology, medical entomology

## Abstract

The Atlantic Forest is a highly diverse biome that is under constant pressure due to human action, resulting in habitat fragmentation and intensifying edge effects, affecting biodiversity. The aim was to study the edge effect and influence of biotic and abiotic parameters on Calliphoridae and Mesembrinellidae communities in Três Picos State Park. Two traps baited using beef liver were placed at each site (n = 5) across 1000 m from the edge toward the interior of the forest, with vegetal characterization at each point. Collections occurred between June 2021 and May 2023, encompassing each season twice. The dipterans were identified taxonomically using a stereoscope microscope with the aid of taxonomic keys, totaling 5476 specimens. Dipteran abundance and species composition were primarily influenced by seasonal variation, while the distance from the forest edge or vegetation structure showed no effect. Abundance peaked during warmer periods, and temperature showed a positive effect on overall dipteran abundance. No species showed a strong association with specific seasons or distance along the edge–interior gradient. These results indicate that, in a relatively continuous and well-preserved forest remnant, edge effects do not lead to significant species loss, and climatic seasonality shapes patterns of dominance and abundance. Our findings highlight the ecological stability of the studied conservation unit and support the use of Calliphoridae and Mesembrinellidae as effective bioindicators. Understanding how dipteran assemblages respond to seasonal and edge-related gradients contributes to the development of cost-effective biomonitoring tools for tropical forest conservation.

## 1. Introduction

The Atlantic Forest is a diverse biome, composed of a mosaic of forests, sandbanks, mangroves, and high-altitude fields, occurring in 17 Brazilian states. Although it currently has only 7% of its original coverage, its significant diversity of flora and fauna, especially endemic species, guarantees that it is a biodiversity hotspot [1,2]. Even so, anthropogenic pressure on this biome is growing alarmingly [3,4], causing habitat loss and fragmentation and highlighting the need to create environmental protection areas [5,6].

The constant anthropogenic pressure and fragmentation experienced by this biome has resulted in amplification forest fragmentation and, consequently, edge effects. Forest edges are defined as transition zones between anthropized habitats and forests, and there may be significant variability in their three-dimensional structure, such as tree stem width, shape, and density, which impacts the quantity and quality of available habitats [7]. These edges usually have a higher incidence of light, greater thermal and wind amplitudes, and lower humidity. These local climate changes affect populations in a variety of ways, including their physiology, behavior, phenology, abundance, geographic distribution, and dispersal. Such changes are noticeable up to 500 m inland [5,8,9]. As a consequence, the dispersion capacity of species decreases, causing population decline, loss of genetic variability, and invasion of exotic species, which are important causes of extinction and loss of native biodiversity [3,5,7,10,11]. Therefore, owing to the adaptations of biota to environmental conditions, such changes can influence and even eliminate species from their habitats and increase the prevalence of other species better adapted to modified environments [3].

The Insecta class is a group with significant diversity and great ecological relevance that occupies various ecological niches [12]. Among them, their ability to act as environmental indicators stands out, as they are small, sensitive to change, and generally have a short and fast life cycle, occurring with high abundances [13]. Population dynamics information of insect fauna can therefore provide data on the quality of the environment, inferring the preservation or degradation of an area caused by natural or anthropogenic sources [3,10,14].

The order Diptera presents great diversity among insects and is found in practically all ecosystems [15,16]. Within this order, Calliphoridae, commonly called the blowfly [17], has a global distribution and comprises more than 1000 species, organized into approximately 150 recognized genera [18]. Maggots from this family can be responsible for both obligate and facultative myiasis, which gives these dipterans great relevance to both animal [19] and human health [20]. Owing to their habit of visiting contaminated substrates, adults are potential carriers of pathogenic agents [21,22,23]. However, they can also be used in a beneficial way. In the medical field, larvae that feed on necrotic tissues in living hosts (necrobiontophages) are used in biotherapy or larval therapy [24]. In the field of forensic entomology, information on their biology is used to assist in criminal investigations, such as the identification of suspects through molecular analysis, body transposition, and, especially, the determination of the postmortem interval [25,26].

With habits highly related to forest environments, Mesembrinellidae, previously a subfamily of Calliphoridae, has been identified as a bioindicator of preserved environments [10,27,28]. Information on this family is still scarce, and even its phylogeny is still debated, having been elevated to the status of a family and ceasing to be a subfamily of Calliphoridae [28,29]. A recent study reorganized this family, unifying several genera with the genus Mesembrinella Giglio-Toss, 1893 [30].

Studies in Atlantic Forest fragments in the state of Rio de Janeiro, as those from the Tijuca National Park [27] and Tinguá Biological Reserve [3,10], have recorded a great amount of data of the Calliphoridae and Mesembrinellidae species that are characteristically asynanthropic, rarely recording species known for their high synanthropy [27].

Studying the dipterans of Calliphoridae and Mesembrinellidae in this remnant of the Atlantic Forest will provide a better understanding of their diversity at this site. In addition, analysis of the responses of these insects to biotic and abiotic factors that influence their distribution could broaden our knowledge of the behavior of the species recorded in this environment. Thus, we hypothesized that (i) the diversity, composition and abundance of Calliphoridae and Mesembrinellidae vary predictably across seasons and along the forest edge–interior gradient, mediated by biotic (vegetation structure) and abiotic (climatic) factors; and (ii) forest-specialist and asynanthropic species are more strongly associated with interior forest conditions, whereas generalist or synanthropic species are favored under edge-related conditions.

## 2. Materials and Methods

**Figure 1:**
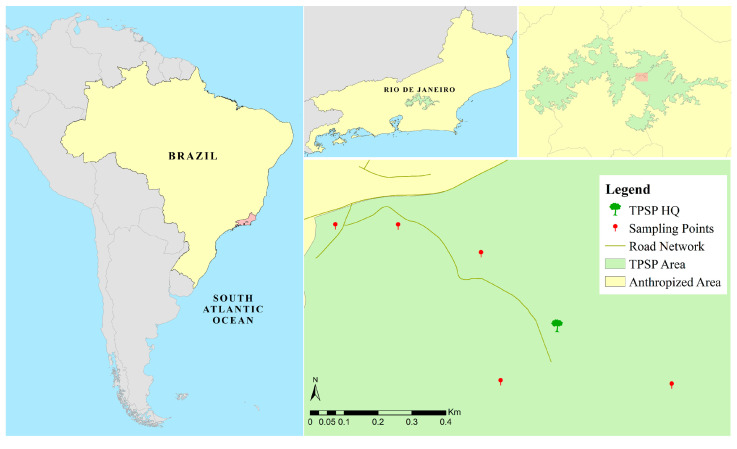
This study was carried out in the Três Picos State Park, located in Cachoeiras de Macacu, Rio de Janeiro, Brazil. Five sites were sampled, in a gradient from the edge toward the interior of the forest, at the distances of 0, 200, 400, 700, and 1000 m. Source: Designed by the authors using ArcGis 10.7.1. Shapefiles obtained from open access sources IBGE (https://www.ibge.gov.br/geociencias/cartas-e-mapas/bases-cartograficas-continuas.html (accessed on 25 November 2025) and gov.br (https://dados.gov.br/dados/conjuntos-dados/unidadesdeconservacao (accessed on 25 November 2025).

Located in Rio de Janeiro, Brazil, the Três Picos State Park (TPSP) (Figure 1) encompasses around 58,790 ha in five cities and is the biggest conservation area in the state, composing the Atlantic Forest ecological corridor. It presents a great diversity of fauna and flora; however, it faces anthropic pressure from illegal activities, urbanization, and wildfires [2].

### 2.1. Sampling Methods

Collections were carried out quarterly, starting in the winter of 2021 (June) and ongoing for two years, until the autumn of 2023 (May) to cover possible seasonality for the taxa of interest. The edge effect was assessed through collections carried out over a gradient of 1000 m from the edge toward the interior of the forest, totaling five sampling points (0, 200, 400, 700, and 1000 m, Figure 1, Table 1), in areas close to the town of Cachoeiras de Macacu. In order to measure the three-dimensional structure and infer the habitat quality and niche availability, the sampling points were characterized via the following vegetation parameters, considering the vegetation within a 3 m radius from each trap: circumference at breast height (CBH); density and richness of vegetation within a radius of 3 m from the traps, considering those with CBH greater than 5 cm; leaf density of shrubs, using a 1 m ruler extended at the 4 cardinal points from the trap site, at a height of 1.5 m, and quantifying how many leaves it touched; and canopy cover, using the Canopeo application (Oklahoma State University) to measure this value via photographs, which were always taken from the same height (1.5 m), oriented toward the north, and at approximately the same time.

Two traps following the model described by Mello et al. [31] were exposed at each sampling point for 48 h, at least 5 m apart and 1.5 m away from the ground. In this way, 10 traps were used per period, totaling 80 traps. The bait used was 300 g of preserved beef liver per trap, bought from a butcher and kept frozen until 24 h before each sampling, when they were moved to the fridge to defrost. After collection, the samples were moved to polyethylene containers and properly identified. The collected specimens were euthanized using absorbent cotton soaked with ethyl alcohol + ethyl acetate solution. The containers containing the samples were transported to the Laboratory for Diptera Studies at the Universidade Federal do Estado do Rio de Janeiro (LED-UNIRIO), where they were kept at −5 °C.

For taxonomic identification, the insects were defrosted and dried using heating light and absorbent paper. The identification of the material consisted of a previous sorting to separate Calliphoridae and Mesembrinellidae from other insects on the basis of morphological characteristics. The dipterans from these families were then pinned, and the species were identified by their morphological characteristics with the aid of a stereoscope, following the taxonomic keys by Mello [32] and Kosmann et al. [33], with updates from Whitworth and Yussef-Venegas [30]. The material was pinned and sent to the entomological collections of the LED and the National Museum (Universidade Federal do Rio de Janeiro) and are awaiting the assignment of voucher numbers, and the remaining material was placed in entomological envelopes and stored in the LED collection. Once confirmed, these numbers will be made publicly available and included in the Supplementary Material, ensuring long-term preservation, traceability, and accessibility for future research. The data on abundance was tabulated using Microsoft Office Excel 2019, recording the period and site of each sample, for further analysis using RStudio (2025.09.2).

Data for the environmental parameter of temperature (°C), humidity (%), and rainfall (mm) from the sampling days were obtained from the National Institute of Meteorology’s Database (BDMEP: http://www.inmet.gov.br/, accessed on 11 October 2023), referring to the meteorological station of Salinas, Nova Friburgo (A624), the closest station located ≅14 km from the sampling sites.

### 2.2. Statistical Analysis

A Coleman curve was produced in order to evaluate whether the sampling effort was enough to reflect the local community. The indices of richness (S), diversity (Shannon–Wiener index, H’), dominance (inverted Simpson index, 1 − D), and evenness (Pielou index, J’) were used to describe the diversity of the species, in addition to Jaccard dissimilarity for the comparison of samples, with measurements of the effects of turnover and nestedness (beta–multi). Next, Jaccard distance (vegdist function, vegan package) cluster plots were produced to illustrate these findings.

To investigate the factors that influence fly abundance, Generalized Linear Mixed Models (GLMMs) with negative binomial distribution (glmmTMB) were adjusted. Due to the strong collinearity between seasonal, climatic, and spatial variables, the analyses were conducted in two sets of independent models. The first evaluated the spatiotemporal variation in abundance as a function of year, season, and collection point. The second evaluated the influence of environmental and structural variables of vegetation, using only continuous predictors. The models were structured in two sets to avoid severe collinearity between variables: GLMM1—spatiotemporal model, evaluating the influence of the year, season, and point of collection on the total abundance of flies, including species as a random intercept effect to control for interspecific heterogeneity: Abundance ∼ Year + Season + Point + (1∣Species). This model allowed the evaluation of seasonal and interannual patterns, as well as spatial variation between points, without the bias caused by collinearity with continuous environmental or structural variables. GLMM2—environmental and structural model, which evaluated the effects of environmental and vegetation variables on fly abundance, using only continuous predictors (temperature, relative humidity, precipitation, PAC, leaf density, tree density, and canopy cover) and again including species as a random effect: Abundance ∼ Temperature + Humidity + Rainfall + CBH + TDens + LDens + Canopy + (1∣Species). This model investigated the environmental and structural drivers of abundance, separately from spatiotemporal variation.

Finally, a diversity analysis of additive diversity partitioning and hierarchical null models (adipart) was performed to evaluate the variation in richness and diversity among the different scales sampled. For all analyses, a significance index of 5% (α = 0.05) was considered.

## 3. Results

**Figure 2:**
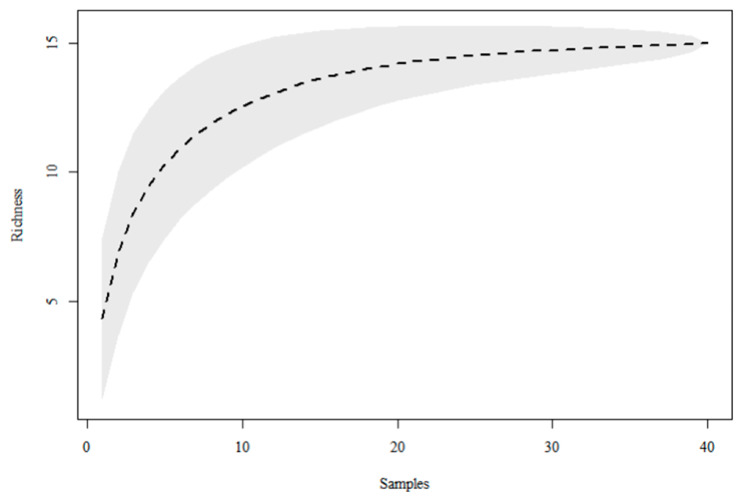
Coleman collector’s curve illustrating the sampling effort of Calliphoridae and Mesembrinellidae collected at Três Picos State Park, Cachoeiras de Macacu, Rio de Janeiro, between June 2021 and May 2023.

This study resulted in the sampling of 5476, totaling 15 species from the Calliphoridae and Mesembrinellidae families (Table 2) in the TPSP. Among these, the Calliphoridae family represented 77.1% of the dipterans, encompassing five species, whereas the Mesembrinellidae family (22.9%) included ten species. The Coleman collector curve demonstrated that the sampling effort was sufficient in capturing the community of these dipterans in the region, with the curve approaching an asymptote (Figure 2).

### 3.1. Characterization of the Sampling Sites

The characterization of the vegetation (Table 3) parameters of the sampled points revealed that the highest canopy cover was observed at the 700 m (74.47 ± 6.31%) and 0 m (72.10 ± 7.91%) points. The smallest canopy covers were observed at the points at 200 m (60.70 ± 28.17%) and 1000 m (61.61 ± 12.81%) (Table 3). The highest leaf density was recorded at the 1000 m point (33 touches), whereas the lowest leaf density was observed at 0 m (14 touches). The highest PAC measurement was 44.36 ± 43.79 cm at the 1000 m point, whereas the lowest observed measurement was 27.20 ± 26.19 cm at the 200 m point. The highest values of density and plant richness were recorded at the point at 1000 m, with 14 plants of 12 species, whereas the lowest records were observed at 400 m, with 7 plants of 7 species.

The first point sampled (0 m) was located near the entrance to the park. The site resembled a valley close to the course of the Macacu River. The second point sampled (200 m) was located near a clearing and a hillside, making it a more open area. The innermost point, at 1000 m, which was also located near a watercourse, experienced recent disturbances as a result of a strong storm, resulting mainly in the fall of a large tree. As a result, the canopy cover at this point has decreased, and the vegetation in the lower strata has begun to thicken. Importantly, the points were chosen according to the trails for easy access, and sometimes, the leaf density measurement was reduced in a certain direction because it was the direction of origin, which was purposely less dense to facilitate location and access to the points. The 400 and 700 m points, on the other hand, reflected what was expected.

### 3.2. The Influence of Biotic and Abiotic Factors on the Abundance of Calliphoridae and Mesembrinellidae

The TPSP is located in a tropical region with two well-defined climatic seasons: summer, with high temperature and rainfall, and winter, with milder temperatures and low rainfall. Spring and autumn are predominantly similar to these periods. According to the unit’s management plan, the annual rainfall is greater than 2000 mm, and the average temperature varies between 18 and 26 °C in summer and between 10 and 18 °C in winter. As expected, summer was the period with the greatest abundance of dipterans of the families of interest, with 2920 individuals, whereas the lowest capture was observed in the winter, with only 477 individuals (Table 4).

**Figure 3:**
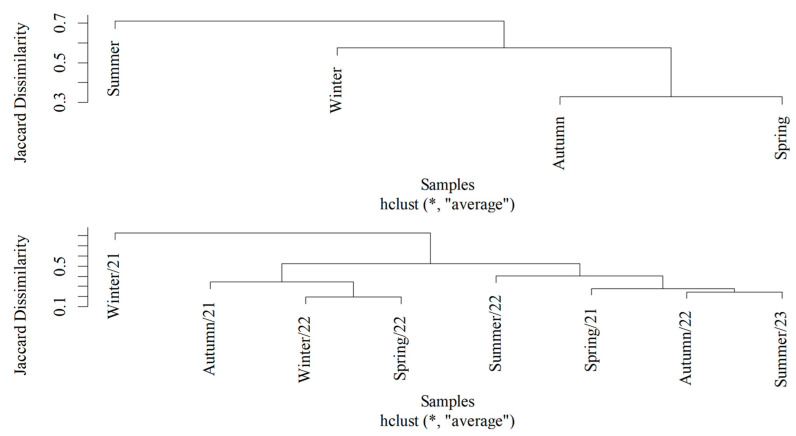
Jaccard dissimilarity cluster of Calliphoridae and Mesembrinellidae communities collected in different climatic seasons (autumn, winter, spring, and summer) in Três Picos State Park between August 2021 and May 2023. (* Jaccard Dissimilarity Matrix for the abundance of Calliphoridae and Mesembrinellidae between climatic seasons).

The diversity analysis revealed lower richness during autumn (S = 9) than during the other seasons (S = 12) (Table 5). The highest diversity was observed in winter (H’ = 1.535), despite the lower abundance (n = 477), which was the most equidistributed community (J = 0.618) and, therefore, had lower dominance (D = 0.293). The opposite was observed for spring: its diversity was the lowest recorded (H’ = 1.006), with high dominance (D = 0.532) and low equity (J = 0.405). The Jaccard similarity index between the stations presented a value of 0.455, with the substitution (turnover) contributing 0.348 and the nestedness 0.107. The seasons that were most similar were autumn and spring (Jac = 0.330), whereas summer was the period with the greatest distinction (Figure 3).

Some species appear to show variation in abundance along the gradient or are more abundant at certain sampling points. *Laneella nigripes* Guimarães, 1977, for example, showed a gradual increase in abundance as collections moved away from the edge (Table 5). *Mesembrinella bellardiana* Aldrich, 1922, on the other hand, was more abundant at intermediate points. *Hemilucilia segmentaria* (Fabricius, 1805) and *Lucilia eximia* (Wiedemann, 1819) were abundant throughout the gradient.

**Figure 4:**
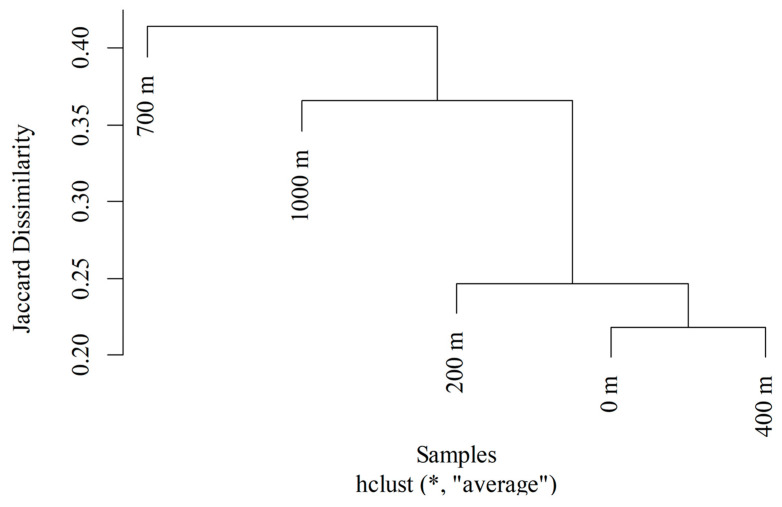
Jaccard dissimilarity cluster of Calliphoridae and Mesembrinellidae communities collected along an edge gradient (0, 200, 400, 700, and 1000 m) in Três Picos State Park between August 2021 and May 2023. (* Jaccard Dissimilarity Matrix for the abundance of Calliphoridae and Mesembrinellidae between distances).

The diversity analysis by collection point (Table 2) revealed that the greatest richness occurred at the innermost point (S = 14), while the point at 700 m had the lowest richness (S = 11). However, this same point had the highest diversity (H’ = 1.540) and evenness (J = 0.642) indices and, therefore, the lowest dominance (D = 0.295). The lowest diversity was observed at the point at 200 m (H = 1.078), whereas the innermost point (1000 m) had the highest dominance (D = 0.503) and, consequently, the lowest equity (J = 0.413). The Jaccard dissimilarity between the points was 0.434, with species turnover contributing 0.319 and nestedness contributing 0.115. Thus, Figure 4 shows that the points that were most similar were those located at 700 and 1000 m (Jac = 0.214) and those located at the edge (0 m) and 400 m from the edge (Jac = 0.218).

The Generalized Linear Mixed Models (GLMMs) corroborate the patterns of temporal variation in fly abundance. The season effect was significant (Table 6), with greater abundance in the summer when compared to the winter (GLMM1, *p* < 0.01), while autumn and spring did not differ significantly from winter. The collection point, however, showed no significant effect, indicating that the abundance does not vary spatially between the sampled sites. To evaluate the fit of abundance models adjusted for the negative binomial distribution, we performed residual diagnoses using the DHARMa package. The Kolmogorov–Smirnov test (simulateResiduals) indicated the normality of the residuals (*p* = 0.055), and the dispersion (testDispersion, *p* = 0.696) and zero-inflation (testZeroInflation, *p* = 0.616) tests did not indicate the presence of significant overdispersion or excess of zeros, respectively. Thus, the negative binomial model was considered to appropriately adjusts the abundance data, being used for subsequent analyses. Despite these differences, no species was associated with a season according to IndVal analysis.

The temperature records showed a wide range, with the lowest temperature recorded at 7.9 °C, occurring in the winter of 2022, and the highest temperature recorded at 27.6 °C, occurring during the summer of 2023 (Table 7). The highest rainfall was recorded during the summer of 2021 (36.9 mm), whereas in the fall of the same year, there was no rainfall. The relative humidity (%) exhibited little variation throughout the collection period, ranging from 85.3% in the summer of 2021 to 76.5% in the summer of 2023.

Among the environmental and structural factors of vegetation, only temperature had a negative effect on abundance (GLMM2, *p* < 0.001) (Table 8). Moisture, precipitation, PAC, leaf density, tree density and canopy cover did not show significant effects. The second adjusted model showed excellent data fit, as indicated by the residual diagnostics performed with the DHARMa package. The Kolmogorov–Smirnov test did not reject the hypothesis of normality of the residuals (*p* = 0.65), indicating good agreement between the observed and expected distribution of the residuals. The dispersion (*p* = 0.82) and zero-inflation (*p* = 0.60) tests also showed no evidence of overdispersion or excess of zeros, respectively.

**Table 8:** Parameters and *p*-values from the Generalized Linear Mixed Model results with negative binomial distribution explaining the abundance of Calliphoridae and Mesembrinellidae collected in Três Picos State Park between August 2021 and May 2023. Predicted variables: temperature, humidity, rainfall, circumference at breast height (CBH), tree density, leaf density, and canopy coverage.

	Estimate	Std. Error	z Value	Pr (>|z|)	
(Intercept)	−0.483	4.723	−0.102	0.919	
Temperature ^a^	0.125	0.032	3.859	<0.001	***
Humidity	−0.010	0.050	−0.201	0.841	
Rainfall	0.007	0.005	1.492	0.136	
CBH	0.001	0.012	0.041	0.968	
TDens	−0.117	0.098	−1.196	0.232	
LDens	0.022	0.027	0.807	0.420	
Canopy	−0.005	0.018	−0.278	0.781	
AIC: 2082.8; Dispersion parameter = 0.508; Random effect variance (species) = 4.883					

^a^ Temperature had a positive effect on abundance, Signif. codes: 0.001 ‘***’.

Taken together, the results indicate that the total abundance of flies is mainly influenced by season and average temperature, but not by collection point or vegetation structure. In addition, the composition of the species presents a significant seasonal change, while the spatial variation between points is negligible. Thus, temporal patterns (seasonality and year) are more important than spatial or structural factors for fly communities in this area.

### 3.3. α, β and γ Diversity in the Context of the Edge Effect

The analysis of partitioned diversity applied to richness revealed that this index, both in relation to the edge gradient (α.1) and the seasons (α.2), was lower than expected, i.e., there was a dominance of few species in each group, with a variation in abundance among the samples, with little influence from the turnover effect (Table 9). The richness β and γ did not differ from what was expected, revealing that the species pool is maintained between the scales, with only variations in abundance. For diversity, a lower-than-expected value was found for the edge gradient (α.1) and higher than expected when analyzed together (β.1), indicating the dominance of some species and small variations that may be caused by the edge effect. No differences were detected between the seasons, and the observed diversity was greater than expected for the park as a whole (γ), suggesting that the edge effect does not interfere with the species pool.

## 4. Discussion

Anthropic impacts directly impact the richness, abundance and composition of dipterans, favoring the synanthropic species, which are adapted to explore and thrive on human-made resources [11]. Sousa et al. [11,34] studied several biomes impacted by cattle ranching and observed the strong influence of human impacts on Calliphoridae, Mesembrinellidae and Sarcophagidae and noted that the higher the heterogeneity of the preserved environment, the higher is the impact after anthropization, due to the diversity of niche loss. However, the authors highlight that the relation between anthropogenic impacts and diversity is not always evident, as the local extinction of forest specialists will be balanced by the arrival of more generalist taxa. When the level of impact is intermediate, it is also possible to observe the increase in richness and diversity, which may be related to the intermediate disturbance hypothesis [35], when species from both the preserved and the impacted areas coexist in the transition area. Although the area studied is placed near the urban area of Cachoeiras de Macacu, and close to a highway, where anthropic impacts are constant and expected to impact the local communities, the community homogeneity along the forest gradient observed in our study indicates a preserved and stable state of this conservation unity. Thus, the edge effect is not acting on the communities of Calliphoridae and Mesembrinelliae in the studied sites.

Mesembrinellidae are exclusively forest-related and Neotropical, with *M. bellardiana* and *La. nigripes* often observed as abundant in forest areas with well-preserved characteristics [36,37], even when the abundance of Calliphoridae is high, indicating that this species has great plasticity [36,37,38]. It is suggested that the Mesembrinellidae fauna varies in constitution from the edge of a forest remnant and that environmental variations can interfere with the capture of these dipterans [3]. According to Gadelha et al. [10], the edge effect results from fragmentation and habitat modifications caused by human activity, resulting in changes in the structure, composition and/or abundance of species, and can extend up to 500 m into the fragment. However, our observations along the edge gradient revealed that the abundance of the species did not vary with distance. Although a distance gradient was made, the collection points do not necessarily reflect a gradient of environmental quality, as observed through the characterization of the vegetation parameters at each point. As discussed by Orlandin et al. [39], the absence of differences observed in our study between the sampled points can be explained by the continuity between the gradients, which does not present any physical barrier for organisms with a dispersion capacity as high as that of the muscoid dipterans. These authors also reported greater dissimilarity between marginal environments and more internal environments, which corroborates the results of this study.

Other studies indicate that the greatest richness and diversity are common in the marginal portions of the fragments, opposing our findings. When studying insect diversity along a forest gradient in Indonesia, Darsono et al. [7] reported greater insect richness and diversity between 0 and 50 m away from the edge. When only the order Diptera was analyzed, however, no difference was observed, which the authors associated with the feeding habits of these insects being the most relevant determining factor for their distribution (presence of flowers, fruits, feces, and carcasses along the gradient). González et al. [8], when studying a fragment of Chaco serrano in Argentina, reported greater richness and abundance at the edge of the environment for the order Diptera, since these places are accessible for resource exploitation both by forest matrices and by anthropized environments. The authors suggest that this area is preferred by these insects for “resource mapping” because along this gradient, they enjoy both forest and anthropic resources (garbage, carcasses from roadkill, among others).

Measurements of vegetation parameters help us understand the preservation and microclimate conditions of each collection point. In more preserved environments, a high degree of stability is expected, allowing some plant species to develop fully and form a denser canopy cover, protecting the area from the wind and direct sunlight. As a result, the vegetation in the lower strata receives less sunlight, which hinders its development. This scenario leads to few well-developed trees with a high CBH, while many seedlings and epiphytes develop, reducing plant and leaf density in the lower strata. In impacted environments, disturbances, such as fallen trees, are expected to create openings in the canopy, allowing more sunlight and wind to enter and the development of more seedlings to more developed stages, increasing plant and leaf density in the lower strata until this space is occupied or, if disturbances are frequent, until the environment is unable to recover [40]. Vegetation structure may influence the composition of local fauna, since the environmental complexity observed in more preserved and heterogeneous sites offers a greater diversity of niches [11,41]. In addition, plant structure alters the local microclimate, such as maintaining local humidity through evapotranspiration [6]. Therefore, by identifying the relationship between a species and the characteristics of the vegetation, we can indirectly infer this relationship to shading, warmer temperatures, and more humid environments, among other possibilities. These dipterans also act as pollinating agents, as in the case of species that have evolved to exude odors similar to decomposing organic matter, making them attractive; therefore, their distribution can be affected by the presence of this specialized flora [6,42,43]. Some authors also report the preference of Mesembrinellidae for decomposing plant organic matter, which may determine their distribution on the basis of the availability of resources such as decomposing fruit [44,45]. However, the vegetation parameters evaluated in our study did not show significant influence on the abundance of these dipterans, which suggests that vegetation structure alone was insufficient in overriding the effects of dispersal and seasonal climatic parameters.

The analysis of diversity by collection points seems to be more related to the characteristics of each point, with greater diversity and equity at the points with greater canopy cover, characteristic of a more preserved environment, whereas the points at 200 and 1000 m presented the lowest coverage and the highest dominance. Although the altitude variation between the points is low, it may influence the distribution of these insects; however, a more in-depth study is necessary. Other factors may cause this variation, such as resource availability, but it was not possible to measure these factors during this study. Because they are exclusively forested, Mesembrinellidae are adapted to milder climates and wetter environments. On the other hand, Calliphoridae have an advantage in warmer environments. Therefore, the highest indices of richness, diversity and equity occurred in the winter climatic season. In the warmer seasons, especially during the summer, Calliphoridae presented more favorable conditions and multiplied, increasing dominance and decreasing diversity and equity. The considerable stability of the populations of *M. bellardiana* and *La. nigripes* over the years of collection is notable, indicating the adaptation of these insects to the conditions of the forest environment studied.

Seasonal variation has been reported in several studies, with the greatest capture of these dipterans occurring during the winter and the opposite occurring during the summer, opposing the results of our study. On the other hand, Azevedo et al. [27], in a study in Tijuca National Park, reported the greatest abundance in the summer and the greatest richness and diversity during the winter and spring. When three environments in Rio de Janeiro (forest, sandbank, and mangrove) were studied, Luz et al. [46] also reported greater abundance during the summer months. Figueiredo et al. [47], studying the community of these dipterans in the Botanical Garden of Rio de Janeiro, reported the same pattern. For Monteiro et al. [48], the opposite was observed, with greater abundance in the milder months. These studies suggest that abiotic factors, intrinsic to each season, are the main determinants of the abundance and diversity of Diptera.

The temperature, relative humidity, and rainfall records provided by INMET during the collection period revealed relatively stable variables, despite the high amplitude of the temperature records. As expected, the highest temperatures occurred during the summer seasons, when the highest dipteran abundances were recorded, whereas the lowest temperatures were recorded during the winter seasons [49], when the lowest total abundances were also observed. Humidity was also fairly stable throughout the seasons, whereas rainfall was more variable. These factors are often related to the abundance of species from the Calliphoridae and Mesembrinellidae families [27,34,50]. Our findings show that temperature is a factor that favors the development of these insects, especially Calliphoridae, which exhibit gregarious behavior in their larval stage and have been reported to accelerate their development at relatively high ideal temperatures, whereas relatively low temperatures slow their development [51,52,53]. Humidity is an important factor, as these are organisms whose desiccation can cause death. Rainfall, in turn, has an influence because it affects the flight capacity of these insects. These last two factors can also influence the availability of food and pupation substrate, making it unusable due to a lack or excess of humidity [54]. However, no significant effect was detected in our findings for both variables.

The analysis of diversity partitioned for richness revealed that abundance within the same set of species was the main factor responsible for the variation in diversity along the edge gradient and between seasons through the dominance of a few more abundant species. This variation is evident in the diversity γ, indicating that there is no regional loss, i.e., these patterns indicate that edge effects reorganize dominance hierarchies without eroding the regional species pool. For diversity, the same pattern is observed for the edge gradient, but there is heterogeneity between the points, which can be explained by the mosaic of characteristics of each sample point. However, between seasons, it is possible to observe that the effect of local dominance is diluted.

## 5. Conclusions

Although the abundance and composition of Calliphoridae and Mesembrinellidae communities varied, there was no significant difference in composition or abundance related to the distance or vegetation parameters along the forest gradient of 1000 m. Regarding the diversity indices, the observed richness was greater at the innermost point, which was also the point of greatest dominance, with the turnover effect being the main factor responsible for the dissimilarity between the communities. The point at 700 m showed the greatest diversity and was also the point with the most typical characteristics of a well-preserved environment (high canopy cover, with trees in more advanced stages of development). The highest abundances and richness were observed during the summer collections, which differed from the winter. The greatest diversity occurred during the winter collection, when the lowest abundance was observed. Temperature showed to positively affect the abundance of these communities.

Our finding evidence the preservation of the studied conservation unity and highlight the relevance of constant monitoring. The use of biomonitors is an alternative and low-cost tool for the administration to detect early and react to environmental disturbances, preventing the problem from escalating. Understanding the impacts of the edge effect and how indicator organisms respond to these and other factors can contribute to understanding the importance of these changes and to planning conservation measures, and could help future research on the development of biomonitoring protocols.

## Figures and Tables

**Table 1:** Georeference of the study sites for collection of Calliphoridae and Mesembrinellidae at distances of 0, 200, 400, 700, and 1000 m from the Jequitibá Nucleus in Três Picos State Park, Cachoeiras de Macacu, Rio de Janeiro, between August 2021 and May 2023.

Distance (m)	Latitude	Longitude	Altitude (m)
0	−22.413700°	−42.614583°	284 m
200	−22.413675°	−42.612788°	334 m
400	−22.414380°	−42.610400°	387 m
700	−22.417783°	−42.609783°	422 m
1000	−22.417800°	−42.604883°	436 m

**Table 2:** Absolute and relative abundance of Calliphoridae and Mesembrinellidae species captured at the Jequitibá Nucleus in Três Picos State Park, Cachoeiras de Macacu, Rio de Janeiro, between August 2021 and May 2023.

Taxa	Abundance	
	n	%
Calliphoridae	4217	77.0
Chrysomyinae		
* Hemilucilia segmentaria*	706	12.9
* Hemilucilia semidiaphana*	200	3.7
* Hemilucilia benoisti*	36	0.7
* Paralucilia nigrofacialis*	28	0.5
Luciliinae		
*Lucilia eximia*	3247	59.3
Mesembrinellidae	1259	23.0
Mesembrinellinae		
* Mesembrinella bellardiana*	891	16.3
* Mesembrinella peregrina*	7	0.1
* Mesembrinella semihyalina*	77	1.4
* Mesembrinella currani*	12	0.2
* Mesembrinella quadrilineata*	4	0.1
* Mesembrinella cyaneicyncta*	7	0.1
* Mesembrinella randa*	1	<0.1
* Mesembrinella aeneiventris*	7	0.1
* Mesembrinella purpurata*	7	0.1
Laneellinae		
*Laneella nigripes*	246	4.5
Total	5476	100
Richness (S)	15	
Shannon Diversity (H’)	1.304	
Dominance (D)	0.398	
Evenness (J)	0.482	

**Table 3:** Characterization of the vegetation parameters of the collection points of Calliphoridae and Mesembrinellidae sampled in the Três Picos State Park, Cachoeiras de Macacu, Rio de Janeiro, considering canopy cover, leaf density of shrubs, circumference at breast height (CBH), and density and richness of vegetation, considering plants with CBH > 5 cm.

Parameter		Distance from the Edge (m)				
		0	200	400	700	1000
Canopy coverage (%)	Mean	72.10	60.70	63.66	74.47	61.61
	sd	7.91	28.17	1.95	6.31	12.81
	Min	66.50	40.78	62.28	70.00	42.66
	Max	77.69	80.62	65.04	78.93	69.99
Leaf density (n)		14	19	19	17	33
Circumference at Breast Height (cm)	Mean	37.14	27.20	35.34	20.04	44.36
	sd	56.71	26.19	63.82	10.75	43.79
	Min	7.80	10.10	9.30	9.30	12.00
	Median	9.00	16.45	10.80	16.35	30.15
	Max	>180.00	93.50	>180.00	39.60	>180.00
Vegetation density (n)		10	12	7	10	14
Vegetation richness (S)		8	9	7	7	12

Legend: sd—Standard deviation.

**Table 4:** Absolute and relative abundance of species of the families Calliphoridae and Mesembrinellidae captured in different stations of the Jequitibá Nucleus of the Três Picos State Park, Cachoeiras de Macacu, Rio de Janeiro, between August 2021 and May 2023.

Taxa	Season							
	Autumn		Winter		Spring		Summer	
	n	%	n	%	n	%	n	%
Calliphoridae								
Chrysomyinae								
* Hemilucilia segmentaria*	210	20.3	27	5.7	55	5.3	414	14.2
* Hemilucilia semidiaphana*	716	1.5	3	0.6	10	1.0	171	5.9
* Hemilucilia benoisti*	4	0.4	8	1.7	7	0.7	17	0.6
* Paralucilia nigrofacialis*	-	-	-	-	-	-	28	1.0
Luciliinae								
*Lucilia eximia*	541	52.2	193	40.5	739	70.9	1774	60.8
Mesembrinellidae								
Mesembrinellinae								
* Mesembrinella bellardiana*	177	17.1	162	34	164	15.7	388	13.3
* Mesembrinella peregrina*	2	0.2	5	1.0	-	-	-	-
* Mesembrinella semihyalina*	24	2.3	27	5.7	5	0.5	21	0.7
* Mesembrinella currani*	3	0.3	2	0.4	4	0.4	3	0.1
* Mesembrinella quadrilineata*	-	-	-	-	1	<0.1	3	0.1
* Mesembrinella cyaneicyncta*	-	-	3	0.6	4	0.4	-	-
* Mesembrinella randa*	-	-	-	-	1	<0.1	-	-
* Mesembrinella aeneiventris*	-	-	4	0.8	1	<0.1	2	<0.1
* Mesembrinella purpurata*	-	-	2	0.4	-	-	5	0.2
Laneellinae								
*Laneella nigripes*	59	5.7	41	8.6	52	5.0	94	3.2
Total	1036	100	477	100	1043	100	2920	100
Richness (S)	9		12		12		12	
Shannon Diversity (H’)	1.323		1.535		1.006		1.265	
Dominance (D)	0.347		0.293		0.532		0.412	
Evenness (J)	0.605		0.618		0.405		0.51	

**Table 5:** Absolute and relative abundance of Calliphoridae and Mesembrinellidae species captured at different points of the Jequitibá Nucleus in Três Picos State Park, Cachoeiras de Macacu, Rio de Janeiro, between August 2021 and May 2023.

Taxa	Distance from the Edge (m)									
	0		200		400		700		1000	
	n	%	n	%	n	%	n	%	n	%
Calliphoridae										
Chrysomyinae										
* Hemilucilia segmentaria*	210	20	89	8.0	164	13.4	105	12.2	138	11.2
* Hemilucilia semidiaphana*	15	1.4	7	0.6	40	3.3	74	8.6	64	5.2
* Hemilucilia benoisti*	12	1.1	15	1.3	8	0.7	-	-	1	0.1
* Paralucilia nigrofacialis*	2	0.2	-	-	5	0.4	19	2.2	2	0.2
Luciliinae										
*Lucilia eximia*	575	54.8	748	67.0	663	54.3	441	51.4	850	69.2
Mesembrinellidae										
Mesembrinellinae										
* Mesembrinella bellardiana*	193	18.4	202	18.1	259	21.2	168	19.6	69	5.6
* Mesembrinella peregrina*	-	-	1	0.1	4	0.3	1	0.1	1	0.1
* Mesembrinella semihyalina*	5	0.5	17	1.5	35	2.9	18	2.1	2	0.2
* Mesembrinella currani*	1	0.1	1	0.1	3	0.2	3	0.3	4	0.3
* Mesembrinella quadrilineata*	1	0.1	-	-	2	0.2	-	-	1	0.1
* Mesembrinella cyaneicyncta*	1	0.1	4	0.4	2	0.2	-	-	-	-
* Mesembrinella randa*	-	-	-	-	-	-	-	-	1	0.1
* Mesembrinella aeneiventris*	-	-	1	0.1	1	0.1	4	0.5	1	0.1
* Mesembrinella purpurata*	3	0.3	1	0.1	-	-	1	0.1	2	0.2
Laneellinae										
*Laneella nigripes*	32	3	31	2.8	36	2.9	54	6.3	93	7.6
Total	1050	100	1117	100	1222	100	858	100	1229	100
Richness (S)	12		12		13		11		14	
Shannon Diversity (H’)	1.255		1.078		1.363		1.54		1.09	
Dominance (D)	0.375		0.489		0.36		0.295		0.503	
Evenness (J)	0.505		0.434		0.532		0.642		0.413	

**Table 6:** Parameters and *p*-values from the Generalized Linear Mixed Model results with negative binomial distribution explaining the abundance of Calliphoridae and Mesembrinellidae collected in Três Picos State Park between August 2021 and May 2023. Predicted variables: Season and Site.

	Estimate	Std. Error	z Value	Pr (>|z|)	
(Intercept)	−0.589	0.627	−0.940	0.347	
Season: Autumn	0.192	0.242	0.793	0.428	
Season: Spring	0.033	0.247	0.132	0.895	
Season: Summer ^a^	1.309	0.235	5.573	2.51 × 10^−8^	***
Site: 200	0.048	0.250	0.193	0.847	
Site: 400	0.220	0.255	0.860	0.390	
Site: 700	0.135	0.250	0.539	0.590	
Site: 1000	−0.041	0.254	−0.161	0.872	
AIC: 2076.1; Dispersion parameter = 0.539; Random effect variance (species) = 4.844					

^a^ Difference from winter, Signif. codes: 0.001 ‘***’.

**Table 7:** Temperature (°C), relative humidity (%), and rainfall (mm) measurements of the collection periods of Calliphoridae and Mesembrinellidae in the Jequitibá Nucleus of the Três Picos State Park, Cachoeiras de Macacu, Rio de Janeiro, between August 2021 and May 2023.

Year	Season	Temperature (°C)			Relative Humidity (%)	Rainfall (mm)
		Max	Average	Min		
2021	Autumn	21.7	15.6	10.3	78.0	0.0
	Winter	18.8	13.5	8.0	77.0	0.1
	Spring	16.8	13.0	9.6	82.8	7.3
2022	Summer	23.3	18.7	16.2	85.3	36.9
	Autumn	19.0	14.2	10.7	76.2	4.5
	Winter	18.0	12.0	7.9	80.3	5.3
	Spring	16.4	12.5	8.8	81.5	4.1
2023	Summer	27.6	21.3	16.5	76.5	2.9
Mean		20.2	15.1	11.0	79.7	7.6
Standard deviation		2.7	2.5	2.8	3.1	12.0

Source: National Institute of Meteorology (BDMEP-INMET: http://www.inmet.gov.br/, accessed on 11 October 2023), referring to the Salinas meteorological station, Nova Friburgo (A624).

**Table 9:** Analysis of partitioned diversity (α, β and γ) between the edge gradient, the seasons and the Três Picos State Park, for Calliphoridae and Mesembrinellidae dipterans captured in the Jequitibá Nucleus of the Três Picos State Park, Cachoeiras de Macacu, Rio de Janeiro, between August 2021 and May 2023.

Index		Statistic	SES	Mean	2.50%	50%	97.50%	Pr (sim.)
Richness (S)	α.1	5.725	−10.1226	7.0556	6.825	7.05	7.2888	0.01 *
	α.2	9.5	−4.47386	10.9242	10.375	10.875	11.5	0.01 *
	β.1	3.775	−0.32417	3.8687	3.3225	3.875	4.4137	0.71 *
	β.2	3.1667	1.42388	2.5438	1.8104	2.5833	3.375	0.17 *
	γ	12.6667	−1.95732	13.468	12.6667	13.3333	14	0.13 *
Shannon (H’)	α.1	1.028365	−6.74266	1.188418	1.142387	1.187939	1.2297	0.01 *
	α.2	1.275336	−0.52447	1.285192	1.248655	1.284735	1.3209	0.65 *
	β.1	0.24697	9.19444	0.096774	0.062572	0.098296	0.1258	0.01 *
	β.2	0.003269	−0.68562	0.015848	−0.02064	0.015782	0.0512	0.41 *
	γ	1.278605	−2.95988	1.30104	1.285905	1.301013	1.3153	0.03 *

Legend: statistic: observed value; SES (Standard Effect Size): shows how far the observed deviates from what is expected, in standard deviations; mean: mean expected value from 99 permutations (null model simulations); Pr. (yes): probability that the observed value will be obtained by chance (*p* value). Signif. codes: 0.05 ‘*’.

## Data Availability

All data is made available in the Open Science Framework, available online: https://osf.io/39tcw/ (first data uploaded on 2 October 2025).

## Supplementary Materials

The following supporting information can be downloaded at https://www.mdpi.com/article/10.3390/life16040672/s1.

## Abbreviations

The following abbreviations are used in this manuscript:

TPSP	Três Picos State Park
